# Primary Angiosarcoma of the Breast: A Case Report and Review of Literature

**DOI:** 10.14740/wjon809w

**Published:** 2014-06-25

**Authors:** Manjari Pandey, Mike G. Martin

**Affiliations:** a University of Tennessee Health Science Center and West Cancer Center, Memphis, Tennessee, USA

**Keywords:** Angiosarcoma, Breast, Sarcoma, MRI, Mastectomy, Radiation

## Abstract

Angiosarcoma of the breast (ASB) is a rare but aggressive tumor with very poor prognosis. It is important to recognize this tumor early and to distinguish between primary and secondary ASB. While mammograms frequently miss these lesions, ultrasound and MRI show promise as imaging modalities. In spite of characteristic features described on pathology, misdiagnosis is common, with over 35% tumors initially thought to be benign. We present the case of a 32-year-old woman with a history of bilateral reduction mammoplasty who presented with a non-tender lump in her right breast. After repeated aspirations and biopsies, a diagnosis of primary ASB was made. She underwent bilateral simple mastectomies followed by adjuvant chemo-therapy and radiation. While surgery is the mainstay of treatment, roles of radiation and chemo-therapy are still evolving; we review the literature and discuss the decision pathways for diagnosis and management of this rare tumor.

## Introduction

Angiosarcoma of the breast (ASB) is a rare tumor, accounting for less than 0.05% of all primary breast neoplasms [1, 2]. Primary angiosarcomas occur in women without any known risk factors, while secondary angiosarcomas are seen in the setting of previous breast irradiation or lymphedema (Stewart Treves syndrome) [3, 4]. Compared to secondary angiosarcomas, primary ASB tends to occur in younger women and are associated with higher incidence of distant metastases [5]. There is paucity of data on how to diagnose and manage these tumors.

## Case Report

A 32-year-old woman with no significant past medical history, other than history of bilateral reduction mammoplasty (14 years prior) presented with a non-tender lump in her right breast. She denied any nipple discharge, changes in overlying skin or any other lumps. She was not on any medications, but had an intra-uterine contraceptive device. She reported a history of rectal cancer in her grandmother, but her personal and family history was negative for any other malignancies.

**Figure 1:**
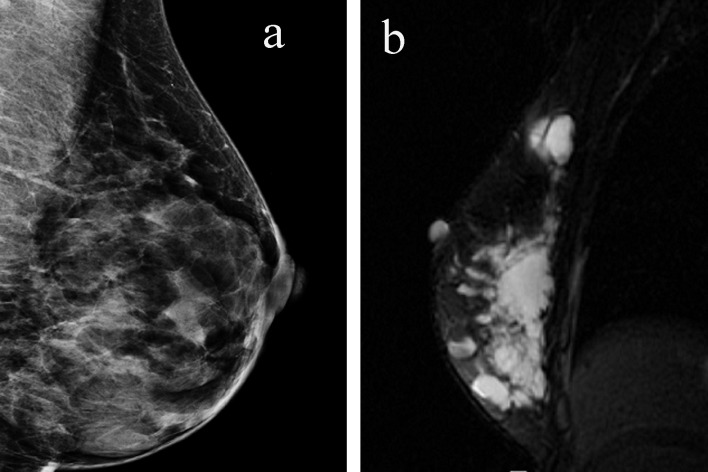
(a) Mammogram reveals multiple cystic lesions. (b) MRI shows the entire right breast is filled with multiple cystic structures with internal loculations.

**Figure 2:**
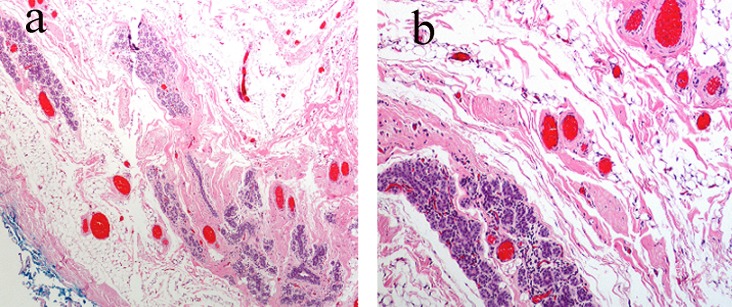
(a) Subtle vascular channels infiltrating the stroma, fat and even ductular tissue of the breast. (b) Higher magnification shows relatively bland vascular lesions but with an obviously infiltrating pattern throughout normal breast tissue.

A diagnostic mammogram showed dense breasts with multiple nodular densities with a well-circumscribed nodular density in the upper outer quadrant of the right breast (Fig. 1a). An ultrasound demonstrated innumerable cysts in both breasts, largest measuring 3.5 × 0.9 cm in size. She then underwent aspiration of the cyst, which revealed clear fluid. She required repeated aspirations; at the third recurrence, the cyst was excised. Gross examination revealed a multiloculated cyst, 1.7 cm in largest diameter. Histopathologically, the breast stroma demonstrated variably increased vasculature, and immunostains were negative for pankeratin but positive for CD31, confirming endothelial lining of the lesion. It was found to be negative for any malignancy and was reported as an angioma. The tissue was sent for a second opinion and a final diagnosis of low-grade angiosarcoma was made (Fig. 2).

MRI of the breast revealed multiple cystic structures in the entire right breast, majority of which contained internal loculations (Fig. 1b). There were no abnormalities noted in the left breast or axillary region. She then underwent a positron emission tomography/computerized tomography (PET/CT) scan which was negative except for some postoperative changes in the right breast.

Given the MRI findings that were highly suspicious for diffuse involvement of right breast with angiosarcoma and the natural history of ASB, the patient elected to undergo bilateral simple mastectomies. Pathology revealed benign breast tissue on the left side, and the right breast showed a poorly-delineated, multi-loculated, thin-walled cystic mass, approximately 5 × 4 × 4 cm in size. Low-grade angiosarcoma was identified in multiple sections from the lower outer quadrant and mid portion of the specimen. Microscopic tumor was present within 0.2 cm of the deep margin, and all other margins were negative. There was no evidence of lymphovascular invasion by the tumor. Post-operative treatment consisted of adjuvant chemo-therapy with paclitaxel on days 1, 8 and 15 of a 4-week cycle, for three cycles. This was followed by radiation therapy, 50 Gy with a boost to the scar with an additional 10 Gy.

## Discussion

ASB is a rare but aggressive neoplasm that affects women of all ages (range from 20 to 87 years) with peak incidence at 45 - 55 years [5, 6]. It is important to distinguish between primary and secondary ASB, and the latter most commonly presents as a violaceous rash and is associated with well-defined risk factors such as prior radiation therapy for breast cancer (relative risk of 15.9) [7] or lymphedema, the Stewart Treves syndrome [8]. Unlike secondary ASB that is usually cutaneous, by definition, primary ASB is a malignant vascular neoplasm that arises within the breast parenchyma, with or without extension into the overlying skin [9]. Primary ASB tends to occur in younger women (aged 20 - 40 years), with most patients presenting with a painless palpable lump. It is frequently missed on mammograms due to dense breasts in young women; this is especially true for low-grade angiosarcomas [10]. Breast ultrasound may show hyper- or hypo-echoic lesions with no acoustic shadowing although these findings are non-specific. On MRI, the tumor tends to have low intensity on T1-weighted and high intensity on T2-weighted images. These findings along with prolonged enhancement on dynamic study and multiple unenhanced areas within the tumor may be specific for ASB [11].

Histologically, ASB can be distinguished from invasive carcinomas (ductal/lobular) due to absence of cytokeratin and positive staining for vascular markers such as CD31, CD34. They need to be differentiated from other vascular tumors such as hemangiomas, angiolipomas and pseudoangiomatous stromal hyperplasia (PASH). Benign lesions tend to be well-circumscribed, with well-formed vascular channels; sarcomas have invasive features, a high mitotic rate and a high Ki-67, while PASH is negative for endothelial markers [9, 12]. However, misdiagnosis is common with a high percentage (up to 37%) of cases initially reported as benign [13, 14]. Painless breast tumors in young women that are highly vascular should be considered malignant unless proven otherwise [15, 16].

Tumors size is an important prognostic factor, with lesions more than 5 cm associated with distant metastasis [16], and the most common site is liver, followed by lung, lymph nodes and bones [16, 17] and even the heart [18]. Rosen et al demonstrated that histological grades are associated with prognosis: grade I (low), grade II (intermediate) and grade III (high) were associated with 76%, 70% and 15% 5-year disease free survival respectively [19]. However, grade has not been uniformly seen to affect survival [6] and a retrospective study of 41 cases showed no correlation between grade and risk of recurrence, metastasis or death [9].

Surgical resection of the tumor remains the mainstay of therapy while the role of multi-modality therapy is not clearly defined. Mastectomy is preferable to breast-conserving therapy and due to the rarity of nodal involvement, routine lymph node dissection is not recommended [20]. A recent paper that included both primary and secondary ASB concluded that breast-conservative surgery did not show a worse prognosis compared to mastectomy [21]. The margin status of the tumor is prognostically important in ASB (as in other sarcomas); positive margins are significantly associated with higher risk of local failure (P < 0.002) [22], although it has not consistently shown to be associated with worse overall survival [6, 22].

The role of adjuvant radiation therapy is generally supported by the literature. In a study of 59 patients with sarcoma of the breast, mastectomy followed by radiation therapy was associated with local failure rate of 13% compared to 34% without radiation therapy. This study could not show a statistical benefit for adjuvant radiation, this was attributed to limited sample size and the authors recommended post-mastectomy radiation for positive margins and large tumor size [22].

In a series reported by Anderson, adjuvant radio-therapy was associated with improved 5-year OS (47% vs. 33%) and recurrence free survival (24% vs. 20%); however, this was not statistically significant (P value 0.28 and 0.64 respectively) [17]. Another study on breast sarcomas showed a favorable outcome for conservative surgery and post-mastectomy radiation; however, only eight of the 78 patients had ASB [23].

The ANGIOTAX study was the first phase II prospective clinical trial of patients with metastatic or locally advanced angiosarcomas that established the role of weekly paclitaxel, ORR of 19% and non-progression rate of 24% at 6 months. However, this study included angiosarcomas of all sites that were not amenable to radiation or curative intent resection [24]. In a retrospective review of primary ASB, use of chemo-therapy was not associated with survival; however, an ORR of 48% was seen in metastatic ASB with combination cytotoxic chemo-therapy (anthracycline/ifosphamide or gemcitabine/taxane) [17]. Silverman et al reported that adjuvant chemo-therapy for patients with ASB results in significantly higher relapse free survival (29.2% vs. 4.4%, P < 0.05) [25]. The demonstration of diffuse expression of HIF-1α, WT-1 and VEGF in primary ASB, suggests that therapies directed against these targets should be evaluated [15].

This is the only reported case with a history of bilateral reduction mammoplasty, although there are case reports describing an association of augmentation mammoplasty and breast implants with various malignant tumors, including ASB [26-28]. However, a comprehensive review concluded that breast implants are not associated with an increased risk or a delay in detection of breast cancer [29]. Like many other reported cases, our patient was initially misdiagnosed as a benign tumor, but due to a high index of suspicion, a second opinion was sought and it clinched the diagnosis. Decision for radiation was based on size of tumor and the relatively narrow margin. The decision for adjuvant chemo-therapy was based on patient preference and the ANGIOTAX study led to the choice of paclitaxel. The patient tolerated the therapy well and was free of disease till l5.5 months after therapy when she was found to have an isolated, biopsy-proven metastasis to her liver. She is now being evaluated for resection of the metastatic lesion.

### Conclusion

Primary ASB is a rare but aggressive tumor associated with a poor prognosis. There are limited data on management of these tumors and it is mostly derived from retrospective studies. Surgical resection with negative margins remains the mainstay of therapy and axillary dissection is not routinely recommended. The role of radiation therapy has been supported by multiple studies; however, data on the use of chemotherapy are sparse and extrapolated from treatment of angiosarcomas of all sites. Our case is the only reported case in literature of primary ASB in a patient with history of reduction mammoplasty and highlights the importance of using ultrasound and MRI in imaging. It further underscores the importance of maintaining a high index of suspicion for angiosarcomas in vascular lesions of the breast.

## Abbreviations

ASB: angiosarcoma of breast
MRI: magnetic resonance imaging
PASH: pseudoangiomatous stromal hyperplasia
HIF-1α: hypoxia inducible factor-1α
WT-1: Wilms tumor-1
VEGF: vascular endothelial growth factor
